# Handoff Tool Improves Transitions from the Operating Room to the Neonatal Intensive Care Unit

**DOI:** 10.1097/pq9.0000000000000695

**Published:** 2023-10-07

**Authors:** Julie B. Gallois, Jessica A. Zagory, Brian Barkemeyer, Michelle Knecht, Lauren Richard, Kathleen Vincent, David Sciacca, Crystal Maise-Dykes, Christy Mumphrey

**Affiliations:** From the * Department of Pediatrics, Division of Neonatology, Louisiana State University Health Sciences Center School of Medicine, New Orleans, Louisiana; † Department of Surgery, Louisiana State University Health Sciences Center School of Medicine, New Orleans, Louisiana; ‡ Children’s Hospital of New Orleans, New Orleans, Louisiana.

## Abstract

**Introduction:**

Standardized handoffs reduce medical errors and prevent adverse events or near misses. This article describes a quality improvement initiative implementing a unique standardized handoff tool and process to transition from the operating room to the neonatal intensive care unit (NICU) at a level-four regional center with many inpatients requiring surgical intervention. Before this project, there was no standardized handoff tool or process for postsurgical transitions. The primary aim was to achieve 80% compliance with completing a structured postoperative OR to NICU handoff tool within 12 months of implementation.

**Methods:**

An interdisciplinary team developed and implemented a standardized NICU postoperative handoff tool and process that requires face-to-face communication, defines team members who should be present, and highlights communication with the family. In addition, the handoff tool compliance and process measures were monitored, evaluated, and audited.

**Results:**

Although not consistent, we achieved eighty percent compliance with the outcome measures using the handoff tool. We did not sustain 80% of appropriate providers present at handoff. In addition, insufficient data assess overall parental satisfaction with the surgical experience. Although improved, the process measure of immediate postoperative family updates did not reach the targeted goal. However, the balancing measure of staff experience and satisfaction did improve.

**Conclusion:**

Implementing a standardized handoff tool and process with an interdisciplinary and interdepartmental collaboration improves critical patient transitions from the operating room to the NICU.

## INTRODUCTION

Hospital-based medical errors are a significant cause of death in patients.^1^ Patients in the neonatal intensive care unit (NICU) are particularly vulnerable to medical errors due to various factors, including size, immaturity, the complexity of care, and prolonged hospitalization.^2,3^ Communication is identified as a significant underlying cause contributing to medical errors.^4^ Transferring patient care presents additional opportunities for communication errors.^2^ Adequate communication and multidisciplinary collaboration for these transitions are imperative to ensure the best care for patients and their families.^5^ Standardized handoffs throughout transition times reduce medical errors, prevent adverse events, and improve communication.^6–8^

Current literature regarding surgical transitions in pediatric patients has found that implementing a standardized handoff reduces medical errors, increases the completeness of information transmitted, improves communication without increasing the length of the handoff, and potentially can decrease the handoff duration.^7,9–11^ Studies on surgical transitions or handoffs in the neonatal population have also demonstrated improved clinical outcomes and reduced communication failures.^7,12^

Children’s Hospital of New Orleans participates in the Vermont Oxford Network’s (VON) internet-based Newborn Improvement Collaborative for Quality (iNICQ). The Vermont Oxford Network is a nonprofit voluntary collaboration of more than 1,400 member centers working together to improve neonatal care worldwide with data-driven quality improvement and research.^13^ From 2018 to 2022, the collaborative focused on improving critical transitions, including any change in an infant’s site of care, plan of care, and/or in the care team.^14,15^ One particularly important transition that can occur for neonatal patients is the transition from the operating room to the NICU.

**Fig. 1.**
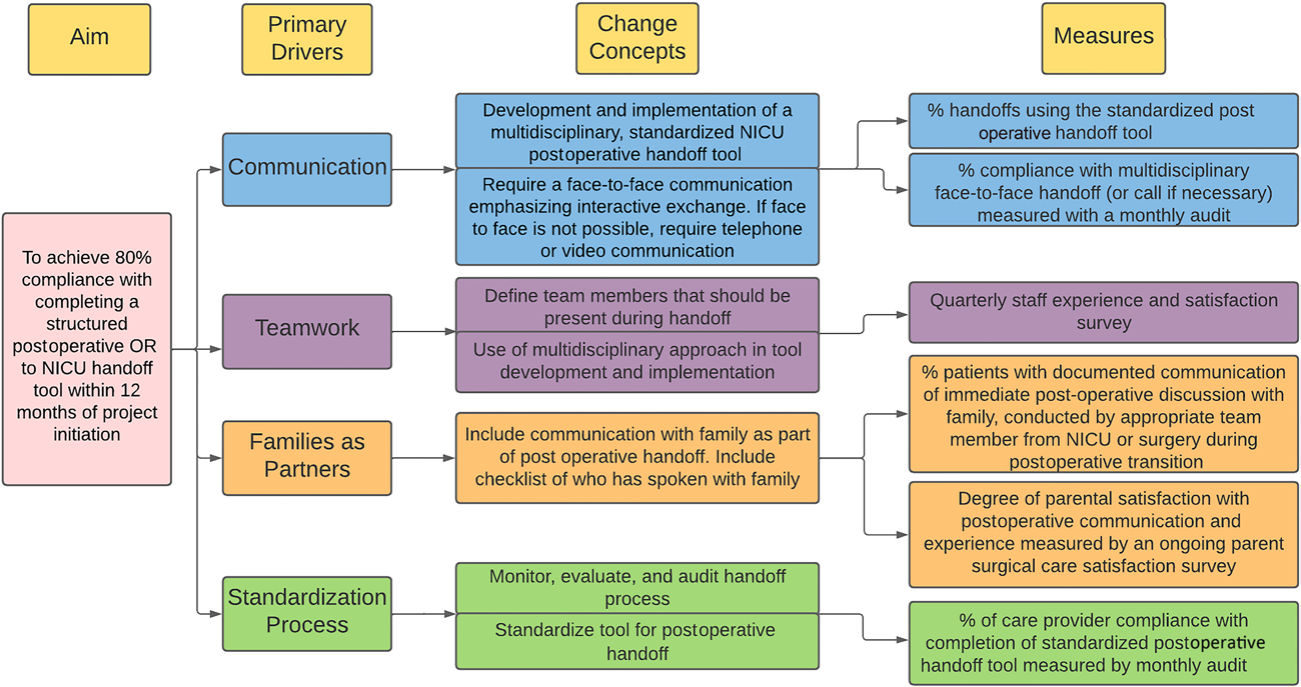
Key driver diagram for improving postoperative transitions in the NICU.

Before this project, there was no standardized handoff tool or process for postsurgical transitions in our unit. This multidisciplinary project aimed to improve postoperative transitions in the NICU by developing, implementing, and evaluating a standardized operating room to NICU handoff tool with a process focused on communication, teamwork, families, and the standardization process as primary drivers (Fig. 1). The primary aim was to achieve 80% compliance with completing a structured postoperative OR to NICU handoff tool within 12 months.

## METHODS

The NICU at Children’s Hospital of New Orleans is a Level IV regional transfer center, and more than half of the patients in this unit have surgical needs. The daily census typically ranges between 30 and 35 inpatients. The number of surgeries in the unit ranged from 5 to 17 per month, with an average of 10 during the study period. Subspecialty surgical services include pediatric general surgery, neurosurgery, otolaryngology, ophthalmology, orthopedics, urology, and interventional cardiology.

On a typical weekday shift, medical staffing in the NICU includes 2 neonatologists, two neonatal nurse practitioners, 1 neonatal fellow, 2 pediatric residents, and 2–5 medical students. There are 16 neonatologists, four neonatal fellows, about 24 annual pediatric residents, and fifteen neonatal nurse practitioners who rotate to staff the unit. The largest surgical subspecialty involved with our patients is general surgery, with five surgical attendings. Registered nurses are assigned 1–3 patients, depending on acuity. Depending on acuity, 2–3 respiratory therapists are always in the NICU. In addition to the staff already mentioned, pediatric anesthesiologists or pediatric certified registered nurse anesthetists participate in surgical handoff. Infants who remain intubated after surgery return immediately postoperatively to the NICU, bypassing the postanesthesia care unit.

**Fig. 2.**
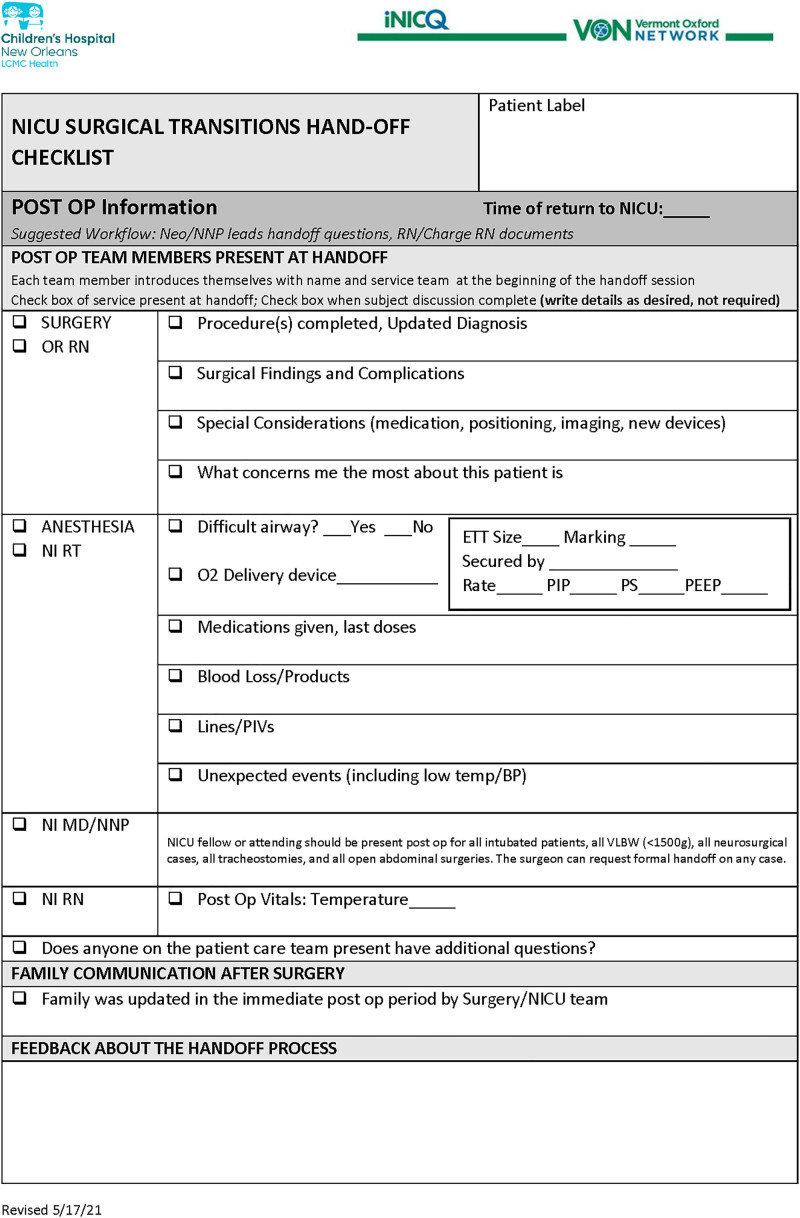
Standardized postoperative handoff tool used during the transition from the operating room to the NICU. OP, operative; O2, oxygen; ETT, endotracheal tube; PIP, peak inspiratory pressure; PS, pressure support; PEEP, positive end-expiratory pressure; MD, medical doctor; NNP, neonatal nurse practitioner; RN, registered nurse; RT, respiratory therapist; OR, operating room).

A multidisciplinary and interdepartmental team assembled as champions and stakeholders for the project. Standardized handoff techniques such as SBAR (Situation, Background, Assessment, and Recommendation) and IPASS (Introduction, Patient, Assessment, Safety Concerns, Background, Actions, Timing, Ownership, Next) are well known to provide a communication framework. Still, these tools were not found to meet the needs surrounding specific communication from surgery to NICU required for optimal patient care and safety.^9^ Our multidisciplinary team developed a standardized NICU postoperative handoff tool (Fig. 2), with use beginning in April 2021.

In creating the tool, surgeons, neonatologists, anesthesiologists, nursing, and respiratory therapists contributed important communication with other team members. This process started in a brainstorming session where essential elements of our unique handoff were identified. For example, surgeons want the team to know “what worries me the most about this patient” in addition to diagnosis and medication guidance. Anesthesiologists want the team to know about difficult airways, and respiratory therapists want to know specific ventilator management strategies used in the operating room. The developed handoff tool outlines a process that requires face-to-face communication, defines team members who should be present at handoff (with guidance based on level of respiratory support, patient weight, and risks of surgical procedure), outlines clinical information to be reviewed and includes communication with the family. Team members communicated electronically and in virtual meetings to contribute input for the handoff tool, and trials of multiple versions of the tool in patient care scenarios continued with ongoing edits.

A 6-question voluntary and anonymous survey measured parental satisfaction (**see survey 1, Supplemental Digital Content 1,** which shows xxx. http://links.lww.com/PQ9/A518). First, 2 questions ask parents to rate their overall experience with their baby’s surgery and their satisfaction with its explanation via a Likert scale. Next, 2 questions ask which physician talked with the parents after the surgery and if updates occurred within 1 hour of the surgery. The final 2 questions are free-text responses asking for improvement suggestions and what parents found helpful for the surgical experience.

Biweekly, the completed handoff tool documentation pages, surgical patient rosters, and parent surveys were analyzed. The primary aim was to achieve 80% compliance with completing a structured postoperative OR to NICU handoff by December 2021. Other outcome measures included the percentage of appropriate providers present at the handoff and parental satisfaction. Process measures included documentation of parental communication immediately postoperative. As a balancing measure, the team surveyed the staff before and after implementing the handoff tool and process (**see survey 2, Supplemental Digital Content 2,** which shows xxx. http://links.lww.com/PQ9/A519).

This study was determined to be a quality improvement project, not research involving human subjects. It was therefore deemed exempt by Louisiana State University Health Sciences Center’s institutional review board.

## RESULTS

### Outcome Measures

**Fig. 3.**
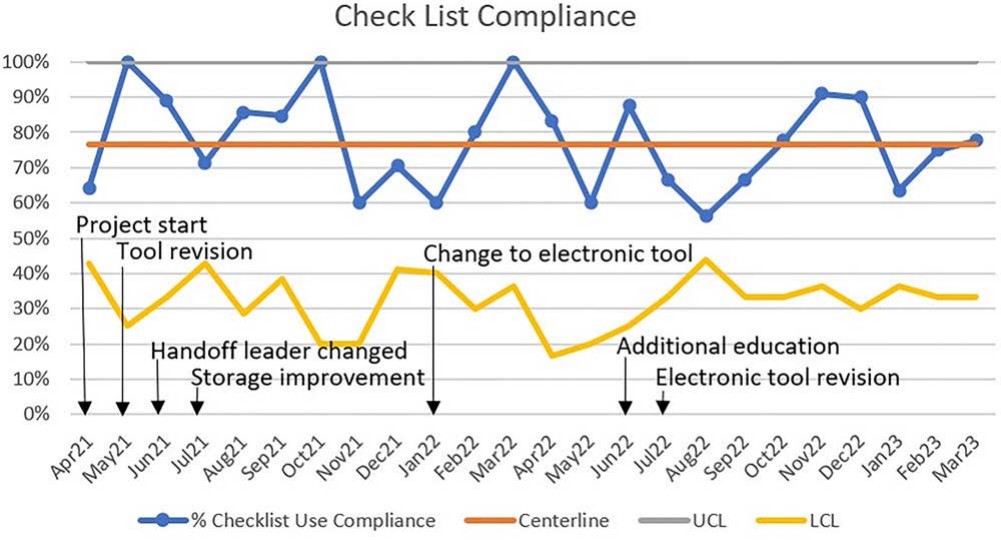
Control chart for the percentage of checklist compliance throughout the study period.

Although we achieved our goal of more than 80% compliance with the handoff tool during most months, we never met control chart rules to justify a centerline shift. Figure 3 shows the results of the control chart for handoff tool compliance. The compliance percentage is calculated by tracking the total checklists completed and the total surgeries in the unit each month. This control chart reflects an in-control process with most points near the middle, a few points at the upper control limits, no points past the upper and lower control limits and no trends, mixtures, stratification, or overcontrol.

**Fig. 4.**
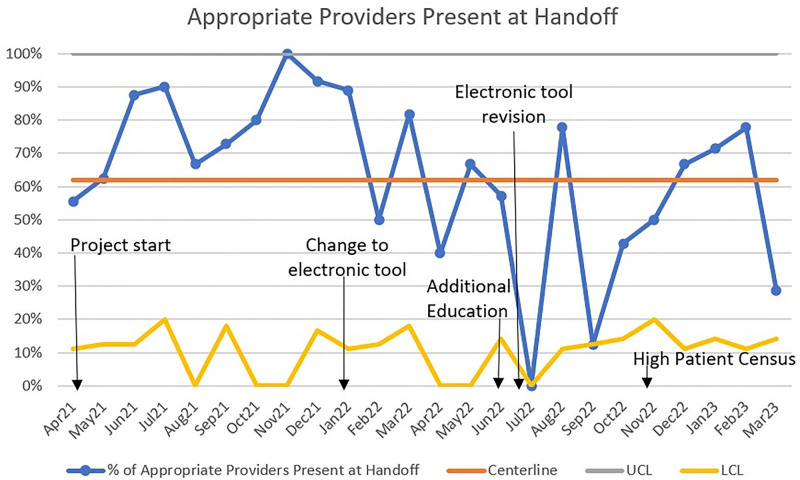
Control chart for the percentage of appropriate providers present at the surgical handoff during the study period.

The results for the percentage of appropriate providers at the bedside during handoff also reached the goal of 80% compliance during the first 9 months of the study, but this improvement was not sustained. The Figure 4 control chart also reflects an in-control process with no trends, mixtures, stratification or overcontrol. However, it does show two special cause points at the lower control limits. There is no data for a pre-implementation baseline for handoff tool compliance or appropriate bedside providers.

Unfortunately, the parent survey had a response rate of only 16%. Although respondents who returned their survey reported their experience as overall positive, the response rate during 10 of the last 14 months of the project is insufficient to be reported as accurate data. In addition, there were no postoperative-specific pre-implementation measures of parental satisfaction as a comparison.

### Process and Balancing Measures

**Fig. 5.**
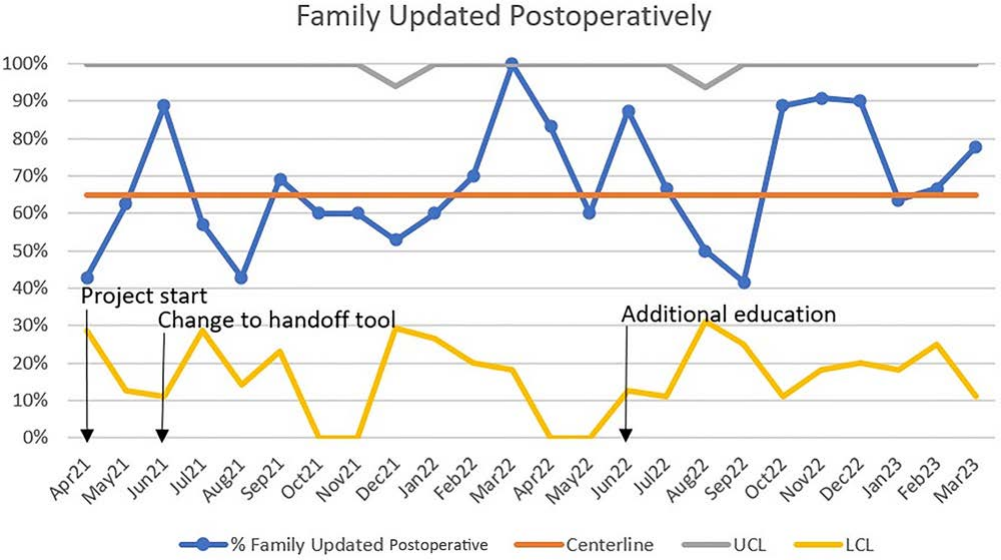
Control chart for the percentage of families updated postoperatively throughout the study period.

The process measure of immediate postoperative family updates did not reach the initial goal of 100% (Fig. 5). However, a trend toward improvement was obtained during 10 of the last 14 months of the project. This measure was calculated from the documentation on the postoperative handoff tool.

The balancing measure for this project assessed staff (physicians, nurses, and respiratory therapists from the NICU, surgery, and anesthesia teams) experience and satisfaction by reviewing pre and posthandoff tool implementation surveys. The results showed that most staff felt a surgical handoff was important (97% pre and 100% post). However, before the implementation of the handoff tool, only 38% of respondents agreed or strongly agreed that a handoff was routinely occurring, and only 21% agreed that the current process provided all the needed information. After implementing the handoff tool, the percentage of respondents that agreed or strongly agreed that handoff was routinely occurring and that it provided needed information rose to 85% and 83%, respectively. Additional responses from the postsurvey showed that about 40% of staff from all units felt that the process could continue to be improved.

## DISCUSSION

This QI study implemented a standardized handoff tool and process to improve communication. The QI team met biweekly to review data and feedback. Using a multidisciplinary approach to develop and revise the tool and handoff process ensured engagement and ownership from the beginning of the project, in agreement with other studies.^5^ Although we have not demonstrated sustained success in all outcome measures of interest, there remains much to learn from single-center experiences in how they work to overcome specific problems.

Incorporating feedback and implementing changes to the handoff tool and process aided in compliance. Making major changes to the handoff tool allowed the achievement of the compliance goal for several months. There were six major versions of the handoff tool. Changes include eliminating the need to write specific provider names, streamlining to a checkbox format, adding brief instructions, adding a specific checkbox reminder to allow time for all care team members to ask clarifying questions and formatting into easy-to-read boxes.

The handoff process changed throughout the project, including storage improvement, expansion of surgical services, transitioning the handoff leader, and refining calls notifying patients’ parents to return to the NICU. The checklist tool that guides handoff was initially electronically stored in the hospital intranet resources and printed as needed. Incorporating nurse feedback, changes to the process included preprinting multiple copies of the paper version of the tool and similarly storing them with other forms on the unit. Changes in the handoff process also included expanding the handoff tool to include all surgical procedures outside of the neonatal unit. Infants undergoing cardiac surgery at our hospital are typically cared for postoperatively in the Cardiac Intensive Care Unit. Patients with post-catheter patent ductus arteriosus closure were not initially included in this handoff procedure, but now they are. As the project progressed, surgery teams refined the timing of their calls to the NICU to report that the patient was returning to the NICU so that team members had time to assemble.

The role of the leader for the handoff changed. Initially, bedside nurses were tasked with leading the handoff procedure. However, the bedside nurses felt that leading the handoff hindered patient care. Therefore, the handoff leader role was transitioned and is now the responsibility of a physician or neonatal nurse practitioner.

In addition to the handoff tool and process changes mentioned above, other methods for increasing compliance with the tool included frequently reviewing the tool during NICU morning huddles when discussing surgical patients for the day and assigning the charge nurse to obtain a copy of the tool when needed. In November and December 2022, compliance decreased when the unit experienced a higher-than-normal census and a staffing shortage. Additional education on the process occurred, which resulted in an improvement in compliance. Communication regarding the handoff tool and process updates occurred in the NICU nursing and division monthly meetings and electronically to other stakeholders as needed.

As the handoff process became a more familiar routine, there was an overall increase in the appropriate providers present at the outset of the handoff. Although specific provider attendance at handoffs is not widely reported in the literature, a recent study found similar results of increased provider attendance at surgical handoffs after implementing a multidisciplinary handoff.^8^ Analysis of the paper version of the checklist for compliance and other measures occurred at the project’s beginning. The paper version of the checklist is now incorporated into the medical provider’s postoperative note in the patient’s electronic health record. After we converted our handoff tool from paper to electronic form, there was a decrease in compliance in later months during the study period. We suspected there was no true decrease in compliance of appropriate providers, but a documentation flaw existed in the electronic reporting version. This flaw could also have created a higher burden on the team’s work and decreased compliance. We updated the electronic documentation to correct potential errors, and the evaluation of this measure continues.

Appropriate providers at handoff were documented at the lower control limit throughout the study. If documentation is accurate, this reflects a decline in the goal. It takes continued reminders to all team members that bedside handoff will occur. Turnover in NICU nursing, monthly resident changes for NICU and surgery, and overall high hospital census could be contributing factors. Neonatologists, surgeons, and anesthesiologists agree that their respective units are busy but not too busy to participate in handoff. Future measures could include assessing the number of handoffs with a complete team present, more specific guidance for surgery residents if they are the accompanying surgeon with the patient for handoff and analysis of which surgical services in particular are not participating. If there is a continued absence of appropriate providers at the bedside, having the handoff be a mandated hospital policy is another avenue.

Families are an integral part of NICU care, and communication with the family is an important part of the handoff tool. However, there is a lack of information regarding the inclusion of updating families in handoff tools with subsequent measurements. The inclusion of updating families in the handoff tool prompts team members to discuss a plan and assign who specifically updates the family.

Many parents cannot be at their infant’s bedside during the surgical transition due to various circumstances, including distance and COVID restrictions at the time of this study. Continued improvement regarding the inclusion of families during surgical transitions remains a project goal. There are plans to continue collaborating with the unit’s child life specialists and family liaisons to optimize parent involvement and input.

The staff survey reveals that handoff occurs more frequently and includes the necessary information. Before the implementation of this project, the routine postoperative surgical handoff was not occurring. Now there is an expectation that communication will occur among all team members at the baby’s bedside.

A remaining challenge is standardizing handoffs for patients who recover in the Post-Anasthesia Care Unit and do not return to the NICU immediately postoperatively, as they are not currently included in our process. Therefore, a future addition to this project involves working with the unit’s surgical team partners to refine the handoff method for those specific patients.

Other future additions to this project include tracking necessary interventions, errors avoided, medication or other orders corrected in real-time, and improved documentation of surgical events (eg, length of bowel remaining post intestinal surgery with relative fistula location).

There were several keys that we think will promote this project’s success. First, interdisciplinary and interdepartmental engagement was imperative. VON and VON quality assessment and improvement projects are familiar to NICU teams but less familiar to those from outside disciplines, such as anesthesia or surgery teams, in the case of this project. However, the desire for optimal patient care is universal. This project had direct patient care staff engagement, input, and support from the most influential leaders in each department and hospital. Other studies have shown success when involving multidisciplinary and specialty input.

Adaptability was also a key factor. Changes were necessary to maximize the handoff tool and the process’s efficiency (eg, streamlining checklists) and compliance. Making small, rapid changes based on staff feedback and real-time data allowed for developing a tailored approach for this important transition from the operating room to the NICU. The hospital NICU quality team plans to track data for at least 1 year and re-activate larger-scale work groups if needed. In addition, the handoff tool and process continue to be monitored and refined to ensure the maintenance is optimal for patient care and the unit’s culture.

## ACKNOWLEDGMENTS

We thank the members of the Children’s Hospital of New Orleans NICU, surgical, and anesthesia teams for their support of this project. We also thank the leaders of the NICUQ collaborative who inspired this project and provided mentorship.

## DISCLOSURE

The authors have no financial interest to declare in relation to the content of this article.

## Supplementary Material
