# Supplementary material for: Handoff Tool Improves Transitions from the Operating Room to the Neonatal Intensive Care Unit
Source: Pediatr Qual Saf. 2023 Oct 7;8(5):e695. doi: 10.1097/pq9.0000000000000695 (PMC10561795; doi:10.1097/pq9.0000000000000695)
Supplement: Supplementary file 1 [file pqs-8-e695-s001.pdf]

NICU Surgery Patient's Parent Survey

As we continue to try to improve care for our babies and the experience of our families, the Children's Hospital Neonatal Intensive Care Unit (NICU) would like some feedback from you on your experiences with your baby's surgery. Participation is voluntary and the answers to this survey are anonymous. When you've completed the survey or if you don't want to complete the survey, please return the form to your baby's nurse.

1. Overall, how would you rate your experience around your baby's surgery?

   □ Very Poor □ Poor □ Neutral □ Good □ Excellent

2. How satisfied were you with the explanation of what procedures or surgeries were done in the operating room to your baby?

   □ Very Unsatisfied □ Unsatisfied □ Neutral □ Satisfied □ Very Satisfied

3. Who talked with your after your baby's procedure? (select all that apply)

   □ Surgeon □ NICU Doctor □ Nurse □ Respiratory Therapist

   □ Other ________________________ □ Not sure who spoke with me

   □ No one spoke with me

4. Were you updated within one hour after your baby's procedure?

   □ Yes □ No

5. What could have been done to improve your NICU surgical experience?

6. What was helpful in dealing with your baby's surgery?
