# Supplementary material for: Handoff Tool Improves Transitions from the Operating Room to the Neonatal Intensive Care Unit
Source: Pediatr Qual Saf. 2023 Oct 7;8(5):e695. doi: 10.1097/pq9.0000000000000695 (PMC10561795; doi:10.1097/pq9.0000000000000695)
Supplement: Supplementary file 2 [file pqs-8-e695-s002.pdf]

The Children's Hospital Neonatal Intensive Care Unit (NICU) along with some of our colleagues in surgery and anesthesia are working on a quality improvement project aimed at improving post-operative transitions in the NICU. We ask that you please complete the following survey regarding your experience and satisfaction regarding neonatal surgical transition of care. The answers to this survey are anonymous and will be used in helping assess the progression of this project.

1. What unit do you work in?
   a. Anesthesia
   b. NICU
   c. Peds Surgery (General or Subspecialty)
   d. Other – allow free text
2. What is your role?
   a. Registered nurse (RN)
   b. Physician (Fellow or Attending)
   c. Neonatal nurse practitioner (NNP)
   d. Certified nurse anesthetist (CRNA)
   e. Respiratory therapist (RT)
   f. Other – allow free text

For the following questions, read the statement and answer on the scale your level of agreement or disagreement with each.

3. Formalized handoff for NICU patients after returning from a surgical procedure is an important part of patient care.
   a. Strongly Disagree
   b. Disagree
   c. Neutral
   d. Agree
   e. Strongly Agree
4. Formalized handoff routinely occurs when patients return from the operating room to the NICU.
   a. Strongly Disagree
   b. Disagree
   c. Neutral
   d. Agree
   e. Strongly Agree
5. The current method of handoff for post-operative patients in the NICU provides all of the necessary information needed.
   a. Strongly Disagree
   b. Disagree
   c. Neutral
   d. Agree
   e. Strongly Agree
6. The current method of handoff for post-operative patients in the NICU is efficient and limits needless information about patient care.

    a. Strongly Disagree
    b. Disagree
    c. Neutral
    d. Agree
    e. Strongly Agree
7. The current method of handoff for post-operative patients in the NICU does not need improvement.
    a. Strongly Disagree
    b. Disagree
    c. Neutral
    d. Agree
    e. Strongly Agree
8. I feel comfortable asking the NICU or OR team questions regarding patient care.
    a. Strongly Disagree
    b. Disagree
    c. Neutral
    d. Agree
    e. Strongly Agree
9. Please feel free to leave comments or suggestions regarding this project or survey.
    a. Free text
